# *Coxiella burnetii* and HIV infection in people experiencing homelessness

**DOI:** 10.1038/s41598-025-09422-z

**Published:** 2025-08-03

**Authors:** Danilo Alves de França, Louise Bach Kmetiuk, Anahi Chechia do Couto, Helio Langoni, Alexander Welker Biondo

**Affiliations:** 1 Department of Animal Production and Preventive Veterinary Medicine, School of Veterinary Medicine and Animals Science, São Paulo State University, Botucatu, SP 18618-681 Brazil; 2 Zoonosis Surveillance Unit, City Secretary of Health, Curitiba, PR 81265-320 Brazil; 3 Graduate Program in Cell and Molecular Biology, Federal University of Paraná, Curitiba, PR 80035-050 Brazil; 4 Department of Veterinary Medicine, Federal University of Paraná, Curitiba, PR 80035-050 Brazil

**Keywords:** Homeless, Q fever, Seroprevalence, Zoonosis, Bacteria, Diagnosis, Public health

## Abstract

This study has investigated *Coxiella burnetii* and HIV infection among the persons experiencing homelessness of São Paulo city, Brazil, and assessed correspondent associated risk factors. A cross-sectional study was carried out with 203 individuals performing serological tests for anti-*C. burnetii* and anti-HIV antibodies. A prevalence of 14.8% (30/203) was found for anti-*C. burnetii* IgG antibodies, with titers ranging from 64 to 1024, while anti-HIV seroprevalence was 6.4% (13/203). No statistical association was found between *C. burnetii* and HIV seropositivity, or between seropositivity and assessed clinical and epidemiological variables. The findings herein highlight the high homelessness exposure to Q fever, possibly influenced by environmental factors such as dust aerosols, stray animal interactions and unsanitary living conditions. To the authors knowledge, this is the first serosurvey of *C. burnetii* in persons experiencing homelessness to date. The study herein has emphasized the importance of public health strategies targeting vulnerable populations, particularly in Brazilian major cities. Further *C. burnetii* surveys should be conducted to establish whether transmission may occur in other persons experiencing homelessness worldwide.

## Introduction

Although the epidemiology of Q fever, caused by *Coxiella burnetii*, remains largely unknown and challenging to be fully understood, recent studies have shown that this pathogen has been capable of infecting a wide range of vertebrate and invertebrate hosts, including livestock, wildlife, companion animals, and arthropods such as ticks^1^. Vulnerable urban populations, particularly people experiencing homelessness, may be exposed and in contact with a variety of vertebrate and invertebrate animals that have been associated with transmission of zoonotic pathogens, including pigeons, rodents, mosquitoes, body lice, and fleas^2^. People experiencing homelessness are often in close contact with stray and unneutered cats and dogs, which may act as *C. burnetii* reservoirs^3^. Although the bacterium shedding during parturition has been well documented in ruminants, few reports have also suggested that companion animals, particularly dogs, may transmit the pathogen during their reproductive events^3,4^. Notably, a report of Q fever outbreak within family members was linked to the birth of an infected dog^4^.

As people experiencing homelessness have mostly lived and slept outdoors, such chronic environmental exposure in urban settings has included excessive sun, rain, dust, pollution, unsafe drinking and eating, and related pathogens^5^. During the COVID-19 pandemics, persons experiencing homelessness living in São Paulo presented 54.7% (111/203) IgG seropositivity, the highest prevalence worldwide at the time^6^. In addition, 47.1% (41/87) of shelter workers were also seropositive, posting airborne exposure also as professional risk. Such high SARS-CoV-2 seroprevalence indicated early and highspeed spreading of homeless people infection, associated with sociodemographic and economic vulnerability. In the case of Q fever, reports indicate that *C. burnetii* spores may be easily transported attached to dust particles, carried by wind and aerosolization travel more than 30 km away^7^. As unsafe food may be another critical factor to *C. burnetii* infection among people experiencing homelessness, with consumption of uninspected or improperly stored food, including discarded animal products such as unpasteurized milk and raw meat as associated risk factors for infection.

The poor homeless living conditions may include a nutritionally deficient and insufficient diet, alcohol and drug abuse, which altogether may lead to stress and immunosuppression, exacerbating infection as observed in other vulnerable populations^8^. In addition, seropositivity for both Q fever and HIV may be particularly alarming, as persons experiencing homelessness have reportedly shown comparative higher HIV seroprevalence and onset, mostly due to unsafe sex and drug injection sharing, leading to syphilis and hepatitis C coinfection, followed by difficult access or incompleteness of antiviral treatment^9^. Although social vulnerability has already been previously associated with Q fever, to the authors knowledge, no study to date has surveyed persons experiencing homelessness^10^.

Studying megacities in developing countries is essential to understand public health dynamics in settings of high population density, social inequalities, and adverse environmental conditions. These locations represent an environment conducive to the circulation of infectious diseases, especially in vulnerable populations, such as people experiencing homelessness, who face multiple risk factors, including exposure to zoonotic pathogens and immunosuppression^11^. Accordingly, the present study aimed to assess anti-*C. burnetii* antibodies in persons experiencing homelessness living in the city of São Paulo, along with potential HIV seropositivity and associated risk factors.

## Results

In overall, the prevalence of anti-*C. burnetii* IgG antibodies among the persons experiencing homelessness in the city of São Paulo was 14.78% (30/203) (95% CI 10.55–20.31). Among the seropositive individuals, 21 presented a titer of 64, 6 a titer of 128, 2 a titer of 256 and 1 a titer of 1024. The prevalence of HIV antibodies was 6.40% (13/203) (95% CI 3.78–10.65). Only in one individual was seropositive for both *C. burnetii* and HIV. Association between serological results and the variables considered as potential associated risk factors for infection was assessed and presented (Table 1). No statistically significant association was found between Q fever seropositivity and analyzed variables, including HIV seropositivity.

**Table 1:** Clinical and sociodemographic factors associated with *C. burnetii* seropositivity in persons experiencing homelessness of São Paulo city, Brazil (2020).

Variables	*C. burnetii*	*C. burnetii*	Crude PR (95% CI)	*P* value	Ajusted PR (95% CI)	*P* value	Total population
	Positive	Negative					
Age (years old)							
30 to 60	22	120	1.0 (ref)		1.0 (ref)		142
< 30	4	25	0.89 (0.33–2.39)	0.818	0.90 (0.36–2.27)	0.831	29
> 60	4	28	0.81 (0.30–2.18)	0.672	0.82 (0.32–2.07)	0.675	32
Ethnicity							
Non-white	17	126	1.0 (ref)		1.0 (ref)		143
White	13	47	0.60 (0.57–1.29)	0.073	1.42 (0.86–2.35)	0.169	60
Raw meat consumption							
No	26	143	1.0 (ref)		1.0 (ref)		169
Yes	4	30	0.77 (0.29–2.05)	0.594	0.94 (0.61–1.45)	0.792	34
Owner of a stray animal							
No	27	145	1.0 (ref)		1.0 (ref)		172
Yes	3	28	0.62 (0.20–1.91)	0.401	0.66 (0.22–1.98)	0.460	31
Rats in the sleeping area							
No	21	119	1.0 (ref)		1.0 (ref)		140
Yes	9	54	0.95 (0.46–2.00)	0.895	1.00 (0.49–2.06)	0.997	63
Symptoms in the past 6 Months*							
No	16	66	1.0 (ref)		1.0 (ref)		82
Yes	14	107	0.59 (0.31–1.15)	0.121	0.60 (0.31–1.19)	0.145	121
Drug use							
No	10	54	1.0 (ref)		1.0 (ref)		64
Yes	20	119	0.92 (0.46–2.07)	0.817	0.97 (0.60–1.58)	0.899	139
HIV seropositivity							
No	29	161	1.0 (ref)		1.0 (ref)		190
Yes	1	12	0.50 (0.07–3.41)	0.483	0.53 (0.07–3.79)	0.531	13

PR: Prevalence Ratio. CI: Confidence Interval.* P* value < 0.05 indicates a statistically significant difference between categories. 1.0 (ref.): reference category used in the regression model.

*Symptoms included fever, myalgia, headache, difficulty breathing, sore throat, and dry cough.

## Discussion

To the authors knowledge, this is the first report of *C. burnetii* serological survey and associated risk factors in persons experiencing homelessness to date. The seroprevalence of anti-*C. burnetii* IgG antibodies identified herein has shown the importance of investigating vulnerable urban populations, who may be exposed to social and environmental conditions favoring infection, particularly by zoonotic pathogens, including *Bartonella* spp., *Rickettsia* spp., *Leptospira* spp., *Toxoplasma gondii*, *Toxocara canis*, *Trypanosoma cruzi* and SARS-CoV-2^6,12–17^.

The study has identified a 14.8% prevalence of anti-*C. burnetii* antibodies and 6.4% of anti-HIV antibodies in the persons experiencing homelessness of São Paulo city. The seropositivity for both *C. burnetii* and HIV was observed in only one out of 203 individuals, with no statistical association of risk factors with *C. burnetii* seropositivity including age, raw meat consumption, presence of rats, drug use and stray pet ownership.

The overall seroprevalence of *C. burnetii* in asymptomatic populations has widely varied, influenced by country, occupation and contact with infected reservoirs^17^. In Brazil, epidemiological surveys indicate an average of 3% prevalence^1^while in Australia, an endemic country for Q fever, the prevalence may reach up to 10% ^2^. The prevalence has also been usually higher in rural and meatpacking workers, due to exposure risk during daily labor, and the highest prevalences recorded to date, as expected, in symptomatic populations^18^.

The seroprevalence herein was higher than 0.9% (8/893) indigenous individuals^8^ and 8% (25/309) forest rangers and zoo workers^19^and lower than 22% (44/200) *quilombola* (former Black slave) and 34% (139/413) female inmates^20^. Proximity to farm animals, intimate contact and isolation, and interaction with wildlife have been associated with *C. burnetii* exposure in Brazil.

The *C. burnetii* ability to survive in dust particles and be transported by wind over long distances may greatly increase the possibility of infection in such a high vulnerability scenario^21^as observed in the present study. In addition, the present study has focused on the human population, with no survey in stray dogs, cats and other animal species with potential homeless interaction. As animal contact may have contributed to the *C. burnetii* exposure^22^such transmission route should be further investigated.

In addition, although a previous study in southern Brazil has shown a higher Q fever prevalence in injectable drug users^23^statistical analysis herein has shown no association of drug use, even with immunosuppressive agents, with *C. burnetii* seropositivity. Although direct contact with pets has apparently not influenced Q fever seropositivity^22^no data was available on the pet reproductive history, and such limitation should be further investigated.

Also, molecular testing could be useful for identifying active *C. burnetii* infections, particularly in symptomatic individuals^24^. However, PCR sensitivity has been limited even in acute cases, and its use in the asymptomatic homeless population herein could be even more restricted due to the low probability of detectable bacteremia^25^. The current recommendation of diagnostic approach in symptomatic patients has been paired serology, as PCR may rarely detect the bacterium in blood samples^26^.

As a limitation, the present study has not included a control group composed of non-vulnerable individuals living in the same urban setting, that may determine whether the observed seroprevalence among people experiencing homelessness was significantly higher than general urban population. Future studies may include comparative groups to better understand potential disparities in exposure.

These findings suggest that people experiencing homelessness, due to their heightened exposure to environmental and social risk factors, may represent a neglected population that could provide early warning signs of zoonotic pathogen circulation in urban areas. This highlights the importance of including this vulnerable population in zoonotic pathogen surveillance efforts and the need to reduce social disparities.

## Methods

### Ethical statement

The present research was approved by the Human Research Ethics Committee, Brazilian Ministry of Health, through the Federal University of Paraná (CAAE: 80099017.3.0000.0102, protocol number 2,512,196) and by the Human Health Ethics Committee of the Municipal Health Department of São Paulo (CAAE: 80099017.3.3004.0086, protocol number 3,366,684). All study participants were informed, voluntary signed a Free and Informed Consent Form, and received their undisclosed results, in compliance with the Brazilian General Data Protection Law.

### Study area

This study was conducted in the São Paulo city (23°33′1″ S, 46°38′2″ W), capital of São Paulo state, located in the southeastern Brazil, and ranked the most populous city and with the largest Gross Domestic Product (GDP) in Latin America, with over 12 million inhabitants^27^. São Paulo presented a high Human Development Index (HDI) (0.805) at the time of survey, with a humid subtropical climate, and average temperatures ranging from 19 °C in winter to 25 °C in summer^27^.

The survey was conducted in partnership with the city team as part of official duties of multi-tasking professionals at the São Paulo City Secretary of Health, known as the “street clinic”, composed by clinicians, nurses, dentists, social workers and psychologists, as part of the Brazilian Unified Health System (SUS) strategy. The street clinic official team provides continuous care to the people experiencing homelessness, keeping medical records and providing health care in a permanent follow-up approach.

The descriptive, cross-sectional study was carried out with the persons experiencing homelessness attended at the Our Lady of Good Childbirth (Nossa Senhora do Bom Parto) Shelter, a non-governmental organization located on western São Paulo, funded by the city hall, and daily receiving around 800 to 1,200 persons experiencing homelessness, providing breakfast, lunch and dinner meals, medical assistance, work activities and leisure options.

### Sample collection

Blood samples from people experiencing homelessness were conveniently collected in a four-day survey in August 2020, as permitted by the City Secretary of Health. A sample size of 203 individuals was calculated using a commercial software Epi Info 7.7.7.6 ^28^, considering an estimated 16,000 persons experiencing homelessness at the time in the city of São Paulo, an HIV infection prevalence of 4.9%, a 95% confidence level, and a precision of 5%. Participants were informed during Shelter activities and invited to voluntarily participate in the study, with blood collection performed by cephalic puncture, conducted by a certified nurse. Samples were placed into tubes without anticoagulant and kept at 25 °C until visible clot retraction. Subsequently, the serum was separated by centrifugation at 3,000 revolutions per minute for 10 min and stored at − 20 °C until processing. During the samplings, researchers ran a clothing drive donation and provided clean clothing to needed.

### Epidemiological data collection

The epidemiological analyses were based on a questionnaire taken from all participants by interview, which included: (1) Demographic profile: gender, self-declared race and age; (2) Social profile: having a pet, raw meat consumption, presence of rats in the sleeping area and use of licit and illicit drugs; (3) Health profile: symptoms related to Q fever (fever, myalgia, headache, difficulty breathing, sore throat and dry cough) in the last six months and diagnosis of HIV. Refusal to partially or totally answer any question was accepted and recorded.

### *Coxiella burnetii* serology

Human serum samples were tested by indirect immunofluorescence assay (IFA), performed with Vero cells infected by *C. burnetii* antigen strain At12, and serum tested in 1:64 dilutions using a conjugated anti-human IgG, based on the protocol previously described by França et al.^27^modified by the use of IgG-specific conjugate. Human patients previously tested at the routine laboratory were used as positive and negative controls. The positive samples were further tested to serial dilutions and titrated according to the last dilution in which luminescence was observed.

### HIV serology

HIV detection was conducted using a chemiluminescent microparticle immunoassay (CMIA) method, employing a commercially available HIV Ag/Ab Combo Reagent Kit (Alinity, Abbott Laboratories, Chicago, IL, USA). Such assay has enabled the simultaneous qualitative detection of HIV p24 antigen and antibodies against HIV type 1 (HIV-1 groups M and O) and/or type 2 (HIV-2) in human serum. The resulting chemiluminescent reaction was quantified in relative light units (RLU). Reactive serology cases were confirmed with a commercially available rapid immunoblot assay (DPP HIV1/2, Fiocruz, Rio de Janeiro, Brazil). Additionally, samples from pregnant women and individuals with HIV were tested for T. gondii IgM using CMIA with a commercial Toxoplasmosis IgM Reagent Kit (Alinity Abbott Laboratories, Chicago, IL, USA).

### Data analysis

Epidemiological questionnaire and HIV seropositivity data were gathered and tested as associated risk factors to *C. burnetii* seropositivity. Univariate analyses were performed using Pearson’s chi-square test to estimate crude prevalence ratios (PRs) and 95% confidence intervals. Variables also were included in a multivariate Poisson regression model with robust variance to calculate adjusted PRs. The most suitable model was selected based on variables with statistically significant associations (*p* < 0.05). All statistical analyses were conducted using SAS Studio 3.81 (SAS Institute Inc., Cary, NC, USA).

The initial missing data in the epidemiological questionnaires were later fully completed by researchers on a specific visit, with daycare shelter database accessed, some persons reinterviewed, information and analyzed variables carefully reviewed. Thus, all participants included in the study herein provided complete responses for the considered variables, used in the statistical analysis for Q fever.

Potential sources of biased and confounding factors were also considered. Multivariate analyses using Poisson regression with robust variance were performed. Thus, potential confounding factors were adjusted in the estimation of adjusted prevalence ratios (PRs).

## Data Availability

The datasets generated during and/or analyzed during the current study are available from the corresponding author on reasonable request.
